# Associations between Coenzyme Q10 Status, Oxidative Stress, and Muscle Strength and Endurance in Patients with Osteoarthritis

**DOI:** 10.3390/antiox9121275

**Published:** 2020-12-14

**Authors:** Po-Sheng Chang, Chi-Hua Yen, Yu-Yun Huang, Ching-Ju Chiu, Ping-Ting Lin

**Affiliations:** 1 Department of Nutrition, Chung Shan Medical University, Taichung 402367, Taiwan; s0746002@gm.csmu.edu.tw (P.-S.C.); s0745011@gm.csmu.edu.tw (Y.-Y.H.); s0745018@gm.csmu.edu.tw (C.-J.C.); 2 Graduate Program in Nutrition, Chung Shan Medical University, Taichung 402367, Taiwan; 3 School of Medicine, Chung Shan Medical University, Taichung 402367, Taiwan; cshy352@csh.org.tw; 4 Department of Family and Community Medicine, Chung Shan Medical University Hospital, Taichung 402367, Taiwan; 5 Department of Nutrition, Chung Shan Medical University Hospital, Taichung 402367, Taiwan

**Keywords:** coenzyme Q10, oxidative stress, muscle mass, muscle function, osteoarthritis, aging

## Abstract

Osteoarthritis (OA) causes oxidative stress. Coenzyme Q10 is an antioxidant that participates in energy production in the human body. The purpose of this study was to investigate the relationships among coenzyme Q10 status, oxidative stress, antioxidant capacity, and muscle function in patients with OA. This case-control study recruited 100 patients with OA and 100 without OA. The coenzyme Q10 status, oxidative stress, antioxidant capacity, muscle mass (by dual-energy X-ray absorptiometry), muscle strength (hand-grip and leg-back strength), and muscle endurance (dumbbell curls, gait speed, chair-stand test, and short physical performance battery) were measured. The results showed that both OA and elderly subjects had a low coenzyme Q10 status (<0.5 μM). Oxidative stress was significantly negatively correlated with muscle function (protein carbonyl, *p* < 0.05). Coenzyme Q10 level was positively associated with antioxidant capacity, muscle mass, muscle strength and muscle endurance in patients with OA (*p* < 0.05). Since OA is an age-related disease, coenzyme Q10 may be consumed by oxidative stress and thereby affect muscle function. Raising coenzyme Q10 in patients with OA could be suggested, which may benefit their antioxidant capacity and muscle function.

## 1. Introduction

Osteoarthritis (OA) is an age-related chronic disease that leads to joint pain, stiffness, and physical impairment [1]. Genetic susceptibility, obesity, and aging are risk factors for OA [2]. In addition to risk factors (obesity or aging), the progression of OA is related to the accumulation of reactive oxygen species (ROS), which may regulate the secretion of cytokines and apoptosis to affect intracellular metabolism in chondrocytes [3]. The excessive production of ROS in patients with OA may cause cartilage matrix degradation and exacerbate the OA process [4,5]. The increased oxidative stress molecules may attack proteins and affect skeletal muscle function in elderly individuals [6,7], and it may increase the risk of sarcopenia in patients with OA [8]. Some studies have shown that the antioxidant status is decreased in patients with OA [5,9]. Antioxidant status or antioxidant capacity can protect cells from oxidative stress damage caused by diseases. However, the association of antioxidant status and muscle function is not clear, especially for patients with OA who have a high risk of limited mobility. Therefore, we investigated the relationships between oxidative stress, antioxidant capacity, muscle mass and muscle strength in patients with OA in this study.

Coenzyme Q10 is an antioxidant nutrient that can act as a free radical scavenger [10]. Coenzyme Q10 also participates in adenosine triphosphate synthesis in the mitochondrial respiratory chain [11], which is important for organs with high energy demands, such as skeletal muscle [12]. Aging decreased cellular bioenergy capacity and may result in a depletion of coenzyme Q10 levels [13]. It is well known that chronic diseases such as cardiac diseases, diabetes, and neurodegenerative diseases may influence the coenzyme Q10 status [14,15,16,17]. To date, no clinical study has investigated the level of coenzyme Q10 in patients with OA. The aim of the present study was to examine coenzyme Q10 status, oxidative stress, antioxidant capacity, and their correlations with muscle function in patients with OA.

## 2. Materials and Methods

### 2.1. Participants and Study Design

The present study was designed as a case-control study. We recruited patients with OA and subjects without OA as the control group. The included participants were ≥40 years old and received standing anterior-to-posterior knee X-ray examination. The diagnosis of OA was made according to the Kellgren and Lawrence (K/L) grade [18]. Participants whose K/L grade was ≥2 were OA, and those whose K/L grade was 0 or 1 were without OA (Non-OA group). The exclusion criteria for both OA and Non-OA groups were as follows: (1) patients with rheumatoid arthritis; (2) the use of glucosamine sulfate, a nonsteroidal anti-inflammatory drug, or hyaluronic acid injection therapy in the past month; (3) the use of other antioxidants or coenzyme Q10 supplements, statins, or warfarin medications; and (4) knee replacement surgery. This study was approved by the Institutional Review Board of Chung Shan Medical University Hospital, Taiwan (CSMUH No: CS2−17095). Each subject provided written informed consent to participate in the study.

### 2.2. Data Collection and Biochemical Analysis

The characteristics of the subjects were recorded from a questionnaire containing age and gender. Height and weight were measured, and body mass index (BMI) was calculated. Muscle mass, including whole and trunk muscle mass, whole skeletal muscle mass index (WSMI), and appendicular skeletal muscle mass index (ASMI), was measured by dual-energy X-ray absorptiometry (Hologic, ASY−05119, Marlborough, MA, USA). Fasting venous blood specimens were collected in vacutainers with K2-EDTA anticoagulant (Becton Dickinson, Franklin Lakes, NJ, USA) or without anticoagulant. Plasma, serum, and red blood cell (RBC) samples were prepared after centrifugation at 4 °C and 3000 rpm for 15 min. The biochemical data, including albumin, blood urea nitrogen (BUN), creatinine, glutamic oxaloacetic transaminase (GOT), glutamic pyruvic transaminase (GPT), high-sensitivity C-reactive protein (hs-CRP), and total cholesterol (TC) were measured by an automated chemistry analyzer (Beckman Coulter, DxC 800, Brea, CA, USA; Hitachi 7600−110, Tokyo, Japan).

### 2.3. Oxidative Stress and Antioxidant Capacity Measurements

Malondialdehyde (MDA) in plasma was analyzed by the thiobarbituric acid reactive substance method [19]. Protein carbonyl groups in plasma were measured by condensation reaction with 2,4-dinitrophenylhydrazine [20]. The protein content in plasma was analyzed with a bicinchoninic acid protein assay reagent kit (Thermo Scientific, Rockford, IL, USA). Protein carbonyl is expressed as nmol/mg protein. Total antioxidant capacity (TAC) in serum and RBC was measured by a Trolox equivalent antioxidant capacity assay, and the wavelength was set at 730 nm [21].

### 2.4. Coenzyme Q10 Status Measurement

The level of coenzyme Q10 was measured by high-performance liquid chromatography (HPLC) with an ultraviolet detector [22]. The protein in plasma was precipitated by propanol after centrifugation, and methanol was added to the supernatant at the same ratio. After mixing, the liquid was filtered for analysis by HPLC. Mixed methanol and ethanol were the mobile phase. The analysis column was a LiChroCART^®^RP-18 (Merck, Germany), and the wavelength of the ultraviolet detector was set at 275 nm.

### 2.5. Muscle Strength and Endurance Assessments

The upper and lower limb muscle strength was evaluated as the handgrip and leg-back strength, respectively. Handgrip was assessed by a handgrip dynamometer (TAKEI, TKK-5401, Niigata, Japan). The subjects were asked to stand with the dominant hand hanging down and then grip the apparatus as tightly as they could. Leg-back strength was assessed by a back dynamometer (TAKEI, TKK-5402, Niigata, Japan). The subjects were asked to stand straight on the platform of the dynamometer and grasp a bar that was attached to a chain and dynamometer and then lift up with maximal effort. Both the handgrip and leg-back strength tests were performed in duplicated for one minute, and the best value was recorded. For the muscle endurance tests, upper limb endurance was assessed by dumbbell curls of the left hand. Subjects were instructed to flex and extend the elbow to lift a 5 lb dumbbell for 30 s. Lower limb endurance was assessed by the chair-stand test. We recorded the number of times in 30 s that the subjects could stand from a chair and then return to sit in it. The gait speed was assessed by the 6-min walk test. Subjects were asked to walk on a flat road for 6 min to record the distance that the subjects walked. Furthermore, we used the short physical performance battery (SPPB) to assess the physical performance of these subjects, which included a balance test, gait speed test, and chair-stand test [23,24]. Higher SPPB scores mean that subjects have better physical performance. Sarcopenia was defined according to the Asian Working Group for Sarcopenia [23], which was assessed by appendicular skeletal muscle mass index and muscle strength and endurance.

### 2.6. Statistical Analysis

SigmaPlot software (version 12.0, Systat, San Jose, CA, USA) was used for all statistical tests in the present study. The normality of each distribution was analyzed by the Shapiro–Wilk test. The mean ± standard deviation (median) or percentages are shown for descriptive statistics. Student’s *t*-Test or the Mann–Whitney rank sum test was used to understand the differences in demographic data, coenzyme Q10 status, oxidative stress, and antioxidant capacity between the two groups. The differences in categorical variables were examined by using the Chi-square test or Fisher’s exact test. Pearson’s correlation coefficient or Spearman’s rank order correlation coefficient was calculated to examine the correlations between coenzyme Q10 status and oxidative stress, antioxidant capacity, muscle mass, muscle strength, and muscle endurance. The results were considered statistically significant at *p*-value < 0.05.

## 3. Results

### 3.1. Subjects’ Characteristics

Table 1 shows the characteristics of the subjects. Patients with OA had significantly higher values of age and BUN and lower GPT than the Non-OA group (*p* < 0.01). Male OA patients had significantly lower muscle strength and endurance than Non-OA (*p* < 0.05); female OA patients showed significantly lower values of gait speed (*p* < 0.01) and SPPB scores (*p* = 0.01). Because the patients with OA had a significantly higher age than Non-OA patients, we stratified the subjects by age: ≥65 years as the elderly group and 40−64 years as the middle-aged group. Elderly patients with OA had significantly higher values of BMI (*p* = 0.02) and significantly lower levels of GOT (*p* < 0.01) and GPT (*p* = 0.02) than the Non-OA group. Both OA and Non-OA subjects in the elderly group had significantly lower level of albumin (*p* < 0.01), muscle mass (*p* ≤ 0.01), muscle strength (*p* < 0.05), and muscle endurance (*p* < 0.05) than those in the middle-aged group. In addition, elderly OA patients had a significantly higher level of hs-CRP than the middle-aged group (*p* = 0.04). Regarding the level of muscle mass, muscle strength, and muscle endurance stratified by age, patients with OA in the elderly (*p* = 0.07) and middle-aged groups (*p* = 0.01) had significantly lower gait speed than Non-OA.

Male OA patients in the elderly group showed lower handgrip strength (*p* = 0.09), leg-back strength (*p* < 0.01), and gait speed (*p* = 0.07) than their Non-OA counterparts, as did the middle-aged OA males for dumbbell curls (*p* = 0.06) and gait speed (*p* = 0.09). Female OA patients in the elderly group showed a higher value of whole-body muscle mass and WSMI than the Non-OA subjects (*p* = 0.03). However, there was no significant difference in muscle strength or endurance between the OA and Non-OA groups of elderly.

### 3.2. The Coenzyme Q10 Status, Oxidative Stress, and Antioxidant Capacity of the Subjects

The coenzyme Q10 status, oxidative stress, and antioxidant capacity of the subjects are shown in Table 2. Elderly patients with OA had a slightly significantly lower level of coenzyme Q10 than Non-OA subjects (coenzyme Q10/TC, *p* = 0.06). With regard to oxidative stress, middle-aged patients with OA had a significantly higher level of protein carbonyl than Non-OA subjects (*p* = 0.02). After stratification by age, Non-OA subjects in the elderly group had a significantly higher level of protein carbonyl than those in the middle-aged group (*p* < 0.01). Regarding the level of antioxidant capacity, both elderly and middle-aged patients with OA had a significantly higher level of serum TAC than the Non-OA group (*p* < 0.01). After stratification by age, the level of RBC TAC was significantly lower in elderly patients with OA than middle-aged patients with OA (*p* = 0.02).

### 3.3. The Correlations between Coenzyme Q10 Status, Oxidative Stress, Antioxidant Capacity, Muscle Mass, and Muscle Strength and Endurance

**Figure 1:**
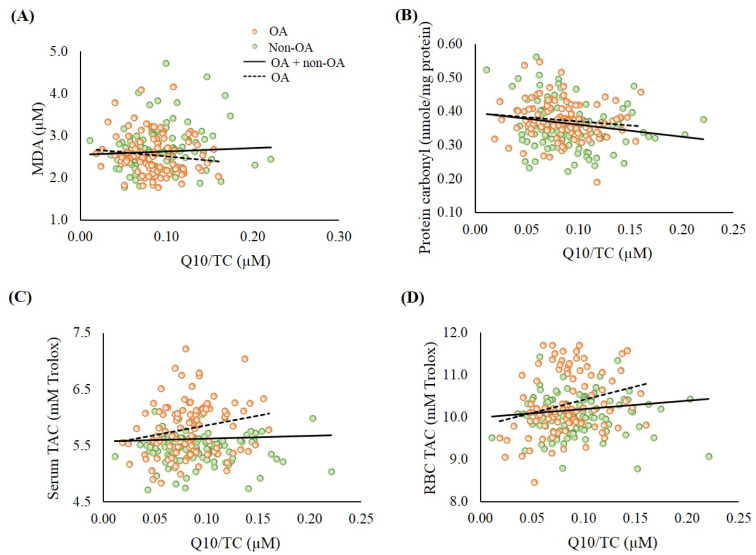
Correlations between coenzyme Q10 status and oxidative stress and total antioxidant capacity. (**A**) Correlation between coenzyme Q10 status and MDA. OA group: *r* = −0.16, *p* = 0.11; OA + non-OA group: *r* = 0.02, *p* = 0.74; (**B**) Correlation between coenzyme Q10 status and protein carbonyl. OA group: *r* = −0.13, *p* = 0.21; OA + non-OA group: *r* = −0.18, *p* < 0.01; (**C**) Correlation between coenzyme Q10 status and serum TAC. OA group: *r* = 0.21, *p* = 0.04; OA + non-OA group: *r* = 0.05, *p* = 0.47; (**D**) Correlation between coenzyme Q10 status and RBC TAC. OA group: *r* = 0.27, *p* < 0.01; OA + non-OA group: *r* = 0.15, *p* = 0.03. MDA, malondialdehyde; OA, osteoarthritis; RBC, red blood cell; TAC, total antioxidant capacity; TC, total cholesterol.

Figure 1 shows the correlations between coenzyme Q10 status and oxidative stress and antioxidant capacity. Coenzyme Q10 status was significantly negatively correlated with oxidative stress (protein carbonyl, *r* = −0.18, *p* < 0.01) and positively correlated with the level of RBC TAC (*r* = 0.15, *p* = 0.03). Additionally, significantly positive correlations were found between coenzyme Q10 status and the levels of serum TAC (*r* = 0.21, *p* = 0.04) and RBC TAC (*r* = 0.27, *p* < 0.01) in the patients with OA. 

**Figure 2:**
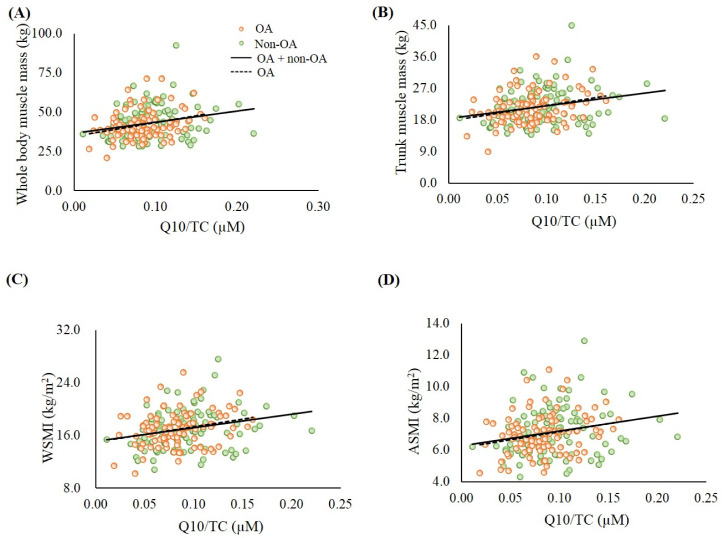
Correlations between coenzyme Q10 status and muscle mass. (**A**) Correlation between coenzyme Q10 status and whole-body muscle mass. OA group: *r* = 0.27, *p* < 0.01; OA + non-OA group: *r* = 0.24, *p* < 0.01; (**B**) Correlation between coenzyme Q10 status and trunk muscle mass. OA group: *r* = 0.24, *p* = 0.02; OA + non-OA group: *r* = 0.24, *p* < 0.01; (**C**) Correlation between coenzyme Q10 status and WSMI. OA group: *r* = 0.26, *p* < 0.01; OA + non-OA group: *r* = 0.26, *p* < 0.01; (**D**) Correlation between coenzyme Q10 status and ASMI. OA group: *r* = 0.24, *p* = 0.02; OA + non-OA group: *r* = 0.24, *p* < 0.01. ASMI, appendicular skeletal muscle mass index; OA, osteoarthritis; TC, total cholesterol; WSMI, whole skeletal muscle mass index.

**Figure 3:**
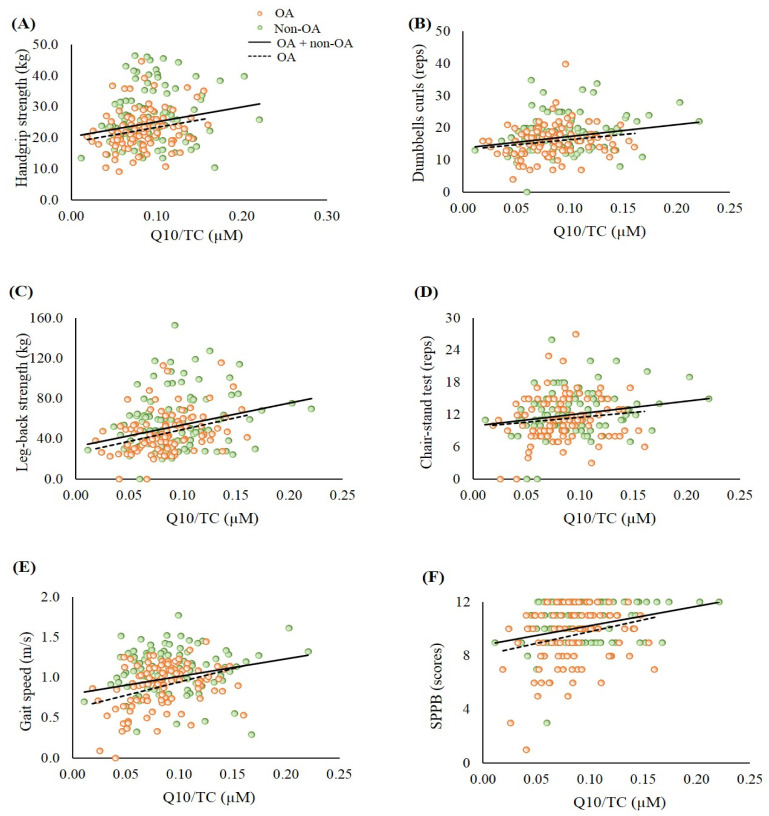
Correlations between coenzyme Q10 status and muscle strength and muscle endurance. (**A**) Correlation between coenzyme Q10 status and handgrip strength. OA group: *r* = 0.24, *p* = 0.01; OA + non-OA group: *r* = 0.20, *p* < 0.01; (**B**) Correlation between coenzyme Q10 status and dumbbells curls. OA group: *r* = 0.24, *p* = 0.02; OA + non-OA group: *r* = 0.22, *p* < 0.01; (**C**) Correlation between coenzyme Q10 status and leg-back strength. OA group: *r* = 0.34, *p* < 0.01; OA + non-OA group: *r* = 0.28, *p* < 0.01; (**D**) Correlation between coenzyme Q10 status and chair- stand test. OA group: *r* = 0.13, *p* = 0.20; OA + non-OA group: *r* = 0.15, *p* = 0.03; (**E**) Correlation between coenzyme Q10 status and gait speed. OA group: *r* = 0.35, *p* < 0.01; OA + non-OA group: *r* = 0.24, *p* < 0.01; (**F**) Correlation between coenzyme Q10 status and SPPB. OA group: *r* = 0.21, *p* = 0.04; OA + non-OA group: *r* = 0.20, *p* < 0.01. OA, osteoarthritis; SPPB, short physical performance battery; TC, total cholesterol.

Next, the correlations between coenzyme Q10 status and muscle mass (Figure 2), and muscle strength and endurance (Figure 3) were analyzed. In the whole sample, the level of coenzyme Q10 was significantly positively correlated with whole body muscle mass (Figure 2A, *r* = 0.24, *p* < 0.01), trunk muscle mass (Figure 2B, *r* = 0.24, *p* < 0.01), WSMI (Figure 2C, *r* = 0.26, *p* < 0.01), ASMI (Figure 2D, *r* = 0.24, *p* < 0.01), handgrip strength (Figure 3A, *r* = 0.20, *p* < 0.01), repetitions of hand dumbbell curls (Figure 3B, *r* = 0.22, *p* < 0.01), leg-back strength (Figure 3C, *r* = 0.28, *p* < 0.01), repetitions of chair-stands (Figure 3D, *r* = 0.15, *p* = 0.03), gait speed (Figure 3E, *r* = 0.24, *p* < 0.01), and SPPB scores (Figure 3F, *r* = 0.20, *p* < 0.01). Similar trends were also found in the patients with OA.

We further examined the correlations between coenzyme Q10 status and oxidative stress, muscle mass, muscle strength, and muscle endurance after stratification by age (Table 3). Coenzyme Q10 status was significantly negatively correlated with oxidative stress (MDA, *r* = −0.21, *p* = 0.07), and positively correlated with antioxidant capacity (serum TAC, *r* = 0.20, *p* = 0.08; RBC TAC, *r* = 0.26, *p* < 0.05), muscle mass (whole body muscle mass, *r* = 0.21, *p* = 0.08; WSMI, *r* = 0.22, *p* = 0.07; ASMI, *r* = 0.26, *p* < 0.05), and muscle strength and endurance (dumbbell curls, *r* = 0.24, *p* < 0.05; leg-back strength, *r* = 0.29, *p* < 0.05; gait speed, *r* = 0.33, *p* < 0.01) in the elderly OA patients. In addition, coenzyme Q10 status was significantly positively correlated with muscle mass in the middle-aged Non-OA group (*p* < 0.05).

### 3.4. The Correlations between Oxidative Stress and Antioxidant Capacity, Muscle Mass, Muscle Strength, and Muscle Endurance

Table 4 shows the correlations between oxidative stress and antioxidant capacity, muscle mass, muscle strength, and muscle endurance. There was a significantly negative correlation between oxidative stress (protein carbonyl) and muscle mass (whole body muscle mass, *r* = −0.23, *p* < 0.01; trunk muscle mass, *r* = −0.26, *p* < 0.01; WSMI, *r* = −0.25, *p* < 0.01; ASMI, *r* = −0.22, *p* < 0.01) and muscle strength and endurance (leg-back strength, *r* = −0.15, *p* = 0.03; gait speed, *r* = −0.33, *p* < 0.01; SPPB, *r* = −0.26, *p* < 0.01). Similar trends were also found in the middle-aged group. On the other hand, antioxidant capacity (RBC TAC) was significantly positively correlated with muscle mass (WSMI, *r* = 0.15, *p* < 0.05) and muscle endurance (gait speed, *r* = 0.27, *p* < 0.01).

## 4. Discussion

This is the first study to investigate the association of coenzyme Q10 status and muscle mass, strength and endurance in patients with OA. In this study, we successfully detected a significantly positive correlation between coenzyme Q10 status and muscle mass, muscle strength, and muscle endurance in patients with OA. OA is an age-related disease that mostly occurs in middle-aged and elderly individuals that may prompt them to face loss of muscle mass and physical function [25,26]. In addition to the level of coenzyme Q10, coenzyme Q10 status may be depleted during aging [27]. Elderly subjects were characterized by impaired coenzyme Q10 status due to a low coenzyme Q10 redox capacity [13]. Indeed, patients with OA had lower coenzyme Q10 status than the Non-OA group, particularly those in the elderly group (Table 2). We also noticed that both the middle-aged and elderly groups suffered from coenzyme Q10 deficiency (plasma coenzyme Q10 < 0.5 μM) [10] in this study. There was a significantly higher proportion of coenzyme Q10 status deficiency in patients with OA than Non-OA (OA, 75% vs. Non-OA, 59%, *p* = 0.02), and the level of coenzyme Q10 dropped with the severity of joint degeneration (K/L grade =2 vs. ≥3, median level of coenzyme Q10: 0.44 vs. 0.39 μM, *p* = 0.04). Previous studies have demonstrated that patients with OA had significantly lower antioxidants such as vitamin C and vitamin E [28,29,30]. Since evidence has indicated that patients with OA suffer from higher oxidative stress [5,31], to neutralize the higher oxidative stress caused by OA, we speculate that coenzyme Q10 may be consumed to slow the progression of OA.

Oxidative stress damage in patients with OA may limit their physical function [6,32]. A result from the FORMoSA project (Bavarian Research Association—Sarcopenia and Osteoporosis) indicated that patients with OA exhibited a higher rate of sarcopenia (OA vs. Non-OA, 9.1% and 3.5%, respectively) [8]. Recently, a Korean report from the Dong-gu study also indicated that joint space narrowing was positively correlated with the muscle strength of OA in both genders [33]. In this study, 17% of patients with OA suffered from sarcopenia, and the prevalence increased with the degree of joint degeneration (data not shown, K/L grade =2 vs. ≥3, prevalence: 12% vs. 23%). Patients with OA may exhibit a decrease in collagen metabolism, mitochondrial impairment in the pathology of sarcopenia, and muscle fiber atrophy, which are associated with oxidative stress caused by the disease [5,34]. As a result, patients with OA in the middle-aged group in this study exhibited significantly higher oxidative stress (protein carbonyl, Table 2), and the level of protein carbonyl was significantly negatively associated with muscle mass, muscle strength, and muscle endurance (Table 4). However, we did not detect a significantly lower antioxidant capacity in patients with OA, except in the elderly OA (Table 2). A decreasing level of antioxidant capacity with joint degeneration was found in the patients with OA (data not shown, K/L grade =2 vs. ≥3, median level of serum TAC: 5.90 vs. 5.57 mM Trolox, *p* = 0.07; median level of RBC TAC: 10.20 vs. 10.00 mM Trolox, *p* = 0.01). The oxidative changes during the progression of OA are complicated. Most patients with OA in the present study were newly diagnosed; we speculated that their higher antioxidant capacity may be caused by compensatory responses to oxidative stress [35,36]. This may also be a reason that the patients with OA did not show a significantly low value of muscle mass in this study (Table 1), but their muscle strength and endurance worsened with the severity of joint degeneration (K/L grade =2 vs. ≥3, *p* < 0.05). Even so, the antioxidant capacity was significantly positively correlated with muscle mass and muscle endurance (Table 4). Thus, increasing the antioxidant capacity of patients with OA may benefit their muscle function as well as decrease oxidative stress during disease progression.

Recent data from two independent population-based cohorts (PopGen control cohort and FoCus cohort) pointed out that coenzyme Q10 status could be a determinant of muscle strength [37]. Del Pozo-Cruz et al. [38] found that coenzyme Q10 status was correlated with functional capacity at an advanced age in community-dwelling people. In the present study, patients with OA had lower muscle mass, muscle strength, and muscle endurance, particularly in the elderly group (Table 1). In addition, significantly positive correlations were found between coenzyme Q10 status and muscle mass, muscle strength, and muscle endurance (Figure 2 and Figure 3). We used the post hoc tests to examine the statistical power of the sample size for the data, and the values for the statistical power of the sample size were 0.8–0.9. It is worth noting that subjects who showed a low muscle strength and endurance score had insufficient coenzyme Q10 status (Figure 3B–E). Since both age and gender are related to muscle function, we examined the correlations between coenzyme Q10 status and muscle mass, muscle strength, and muscle endurance by performing multiple linear regression analyses. After adjusting for age and gender, coenzyme Q10 status was still positively correlated with muscle mass, muscle strength, and muscle endurance (data not shown, *p* < 0.05). The physiological function of coenzyme Q10 is not only to act as an antioxidant, but also to participate in energy production [11]. In this study, we detected a positive correlation between coenzyme Q10 status and antioxidant capacity, particularly in the OA group (Figure 1). As OA patients’ coenzyme Q10 levels went up, they seemed to exhibit a better muscle mass status or muscle performance (Figure 2 and Figure 3). OA is an age- and oxidative-related disease that may contribute to mitochondrial dysfunction [3]. Using mitochondria-targeted antioxidants such as coenzyme Q10 to increase antioxidant capacity may be able to attenuate the progression of OA [39] and further exert beneficial effects on muscle mass and physical performance.

The current paradigm of OA has changed from the concept of a “wear and tear” disease to an inflammation-mediated disease [40]. Coenzyme Q10 has been demonstrated to play a role in OA pathogenesis via pain suppression and cartilage degeneration by inhibiting inflammatory mediators [41]. Although we did not find a relationship between coenzyme Q10 status and inflammation in these OA patients, this might be because our subjects were outpatients who had a stable status. Elderly patients with OA should pay attention their inflammatory status due to their higher level of hs-CRP than middle-aged patients (Table 1).

## 5. Conclusions

In conclusion, patients with OA and elderly subjects suffered from coenzyme Q10 deficiency. Since coenzyme Q10 status was significantly positively associated with antioxidant capacity, muscle mass, muscle strength, and muscle endurance in the present study, we suggest that OA and elderly patients fortify their coenzyme Q10 status, which may benefit their muscle function. Further intervention studies should confirm the causal effects of coenzyme Q10 supplementation on antioxidant capacity and muscle function in patients with OA. Increasing research interest has been focused on myokines such as irisin and myostatin, which may relate to regulate muscle protein synthesis [42,43]; therefore, further study should explore how coenzyme Q10 involved in muscle synthesis.

## Figures and Tables

**Table 1:** Characteristics of the subjects.

Characteristics	OA (*N* = 100)		Non-OA (*N* = 100)			*p* Value ^2^
Age (y)	69.5 ± 9.4 (71.0) ^1^		60.5 ± 9.6 (60.0)			<0.01
BMI (kg/m^2^)	25.3 ± 3.8 (25.6)		24.3 ± 4.3 (24.2)			0.10
Albumin (g/L)	45.0 ± 2.7 (45.0)		45.2 ± 3.1 (45.0)			0.31
BUN (mmol/L)	6.0 ± 2.4 (5.4)		5.1 ± 2.1 (4.6)			<0.01
Creatinine (µmol/L)	76.0 ± 23.9 (71.6)		74.3 ± 23.9 (71.6)			0.58
GOT (U/L)	24.0 ± 5.0 (23.0)		27.1 ± 10.9 (25.0)			0.06
GPT (U/L)	20.9 ± 7.9 (19.0)		27.3 ± 20.7 (22.0)			<0.01
hs-CRP (mg/L)	2.4 ± 7.2 (1.2)		2.2 ± 3.8 (1.0)			0.36
	Male (*n* = 27)	Female (*n* = 73)	*p* Value ^3^	Male (*n* = 40)	Female (*n* = 60)	*p* Value ^3^
Muscle mass						
Whole body muscle mass (kg)	50.5 ± 9.0 (49.5)	38.6 ± 6.4 (38.6)	<0.01	51.5 ± 10.6 (50.9)	38.3 ± 7.4 (36.8)	<0.01
Trunk muscle mass (kg)	25.4 ± 4.6 (24.9)	19.9 ± 3.5 (19.5)	<0.01	25.5 ± 5.2 (25.6)	19.3 ± 3.6 (18.7)	<0.01
WSMI (kg/m^2^)	18.6 ± 2.8 (18.9)	16.3 ± 2.3 (16.4)	<0.01	18.6 ± 2.9 (18.6)	15.8 ± 2.5 (15.7))	<0.01
ASMI (kg/m^2^)	7.9 ± 1.3 (7.9)	6.6 ± 1.0 (6.7)	<0.01	8.1 ± 1.5 (8.1)	6.6 ± 1.3 (6.4)	<0.01
Muscle strength and endurance						
Handgrip strength (kg)	29.3 ± 6.7 (28.7) †	20.2 ± 4.4 (20.1)	<0.01	35.1 ± 7.2 (35.5)	21.2 ± 4.6 (21.5)	<0.01
Dumbbells curls (reps)	16.8 ± 6.5 (17.0) †	15.5 ± 4.3 (15.0)	0.18	21.2 ± 6.1 (20.0)	16.1 ± 4.4 (16.0)	<0.01
Leg-back strength (kg)	65.2 ± 23.8 (61.5) †	38.3 ± 14.0 (38.5)	<0.01	80.0 ± 29.3 (79.3)	42.7 ± 14.8 (44.0)	<0.01
Chair-stand test (reps)	11.5 ± 4.9 (11.0) †	11.1 ± 4.0 (11.0)	0.65	13.1 ± 3.8 (13.5)	12.4 ± 4.0 (12.0)	0.14
Gait speed (m/s)	0.9 ± 0.3 (1.0) †	0.9 ± 0.3 (0.9) †	0.26	1.2 ± 0.3 (1.3)	1.0 ± 0.2 (1.0)	<0.01
SPPB (scores)	9.7 ± 2.1 (10.0) †	9.5 ± 2.2 (10.0) †	0.75	10.9 ± 1.4 (11.5)	10.5 ± 1.5 (11.0)	0.11
Sarcopenia (*n*, %) ^6^	7 (25.9%)	10 (13.7%)	0.23	6 (15.0%)	10 (16.7%)	0.96
	Elderly ^4^			Middle-age ^4^		
	OA (*N* = 74)	Non-OA (*N* = 33)	*p* Value ^5^	OA (*N* = 26)	Non-OA (*N* = 67)	*p* Value ^5^
BMI (kg/m^2^)	24.9 ± 3.7 (25.1)	22.9 ± 4.0 (23.2)	0.02	26.5 ± 3.8 (26.3)	25.0 ± 4.2 (24.8)	0.12
Albumin (g/L)	44.5 ± 2.3 (44.5) *	43.4 ± 3.2 (44.0) *	0.20	46.5 ± 3.4 (46.5)	46.0 ± 2.6 (46.0)	0.53
BUN (mmol/L)	6.2 ± 2.6 (5.4)	6.1 ± 2.9 (5.4) *	0.63	5.5 ± 1.5 (5.4)	4.5 ± 1.2 (4.6)	<0.01
Creatinine (µmol/L)	77.8 ± 24.8 (72.5)	84.9 ± 31.8 (81.3) *	0.18	73.4 ± 19.4 (67.2)	69.0 ± 16.8 (66.3)	0.47
GOT (U/L)	24.1 ± 4.9 (23.0)	29.8 ± 12.4 (27.0) *	<0.01	23.8 ± 5.3 (23.0)	25.7 ± 9.9 (24.0)	0.53
GPT (U/L)	19.9 ± 7.3 (18.0) *	27.5 ± 19.5 (22.0)	0.02	23.7 ± 8.9 (22.0)	27.2 ± 21.4 (22.0)	0.80
hs-CRP (mg/L)	2.9 ± 8.3 (1.3) *	2.0 ± 2.3 (1.1)	0.41	1.2 ± 1.0 (0.9)	2.3 ± 4.4 (0.9)	0.56
Muscle mass						
Whole body muscle mass (kg)	40.1 ± 8.0 (38.7) *	38.8 ± 8.3 (36.5) *	0.24	46.7 ± 9.8 (45.1)	45.9 ± 11.3 (45.7)	0.75
Trunk muscle mass (kg)	20.5 ± 4.2 (19.9) *	19.5 ± 3.9 (18.6) *	0.17	23.9 ± 4.8 (23.2)	23.0 ± 5.5 (23.2)	0.46
WSMI (kg/m^2^)	16.5 ± 2.4 (16.4) *	15.8 ± 2.5 (15.6) *	0.19	18.2 ± 2.9 (17.8)	17.4 ± 3.1 (17.6)	0.26
ASMI (kg/m^2^)	6.8 ± 1.1 (6.7) *	6.6 ± 1.4 (6.3) *	0.25	7.6 ± 1.4 (7.2)	7.5 ± 1.6 (7.4)	0.79
Muscle strength and endurance						
Handgrip strength (kg)	21.2 ± 5.5 (20.6) *	23.1 ± 7.1 (23.9) *	0.19	26.8 ± 7.5 (26.3)	28.6 ± 9.2 (26.7)	0.55
Dumbbells curls (reps)	14.9 ± 5.0 (15.0) *	15.5 ± 5.2 (16.0) *	0.49	18.3 ± 4.2 (18.0)	19.5 ± 5.6 (19.0)	0.57
Leg-back strength (kg)	41.4 ± 16.6 (38.8) *	49.3 ± 25.8 (44.0) *	0.21	57.5 ± 26.8 (53.3)	61.7 ± 28.9 (58.5)	0.61
Chair-stand test (reps)	10.5 ± 4.3 (10.0) *	10.6 ± 3.6 (10.0) *	0.67	13.2 ± 3.7 (13.5)	13.7 ± 3.7 (14.0)	0.64
Gait speed (m/s)	0.85 ± 0.29 (0.90) *	0.96 ± 0.27 (1.02) *	0.07	1.00 ± 0.21 (1.04)	1.15 ± 0.25 (1.17)	0.01
SPPB (scores)	9.1 ± 2.2 (9.0) *	9.8 ± 1.7 (10.0) *	0.14	10.8 ± 1.5 (11.0)	11.1 ± 1.1 (11.0)	0.48
Sarcopenia (*n*, %) ^6^	14 (18.9%)	9 (27.3%)	0.47	3 (11.5%)	7 (10.4%)	1.00
	Male					
	Elderly ^4^			Middle-Age ^4^		
	OA (*N* = 18)	Non-OA (*N* = 10)	*p* Value ^5^	OA (*N* = 9)	Non-OA (*N* = 30)	*p* Value ^5^
Muscle mass						
Whole body muscle mass (kg)	48.3 ± 8.5 (47.0)	46.8 ± 6.9 (45.8)	0.63	54.8 ± 8.7 (53.7)	53.0 ± 11.2 (53.2)	0.56
Trunk muscle mass (kg)	24.4 ± 4.4 (23.6)	23.6 ± 3.3 (23.9)	0.60	27.5 ± 4.7 (26.9)	26.2 ± 5.6 (26.0)	0.52
WSMI (kg/m^2^)	17.7 ± 2.6 (17.7) *	17.9 ± 2.4 (18.3)	0.87	20.4 ± 2.5 (20.4)	18.8 ± 3.1 (19.0)	0.19
ASMI (kg/m^2^)	7.5 ± 1.3 (7.6) *	7.6 ± 1.3 (7.5)	0.92	8.7 ± 1.1 (8.7)	8.3 ± 1.5 (8.2)	0.19
Muscle strength and endurance						
Handgrip strength (kg)	27.0 ± 5.5 (25.8) *	30.5 ± 4.4 (29.8) *	0.09	34.0 ± 6.8 (36.0)	36.6 ± 7.3 (38.3)	0.34
Dumbbells curls (reps)	16.2 ± 7.6 (16.5)	17.7 ± 3.9 (17.0) *	0.41	18.0 ± 3.0 (18.0)	22.4 ± 6.4 (22.0)	0.06
Leg-back strength (kg)	57.7 ± 16.7 (57.8)	79.8 ± 20.1 (78.0)	< 0.01	80.3 ± 29.5 (88.0)	80.1 ± 32.1 (79.8)	0.99
Chair-stand test (reps)	10.7 ± 5.6 (10.0)	10.7 ± 2.5 (10.0) *	0.77	13.1 ± 2.8 (13.0)	13.9 ± 3.9 (14.0)	0.34
Gait speed (m/s)	0.9 ± 0.4 (0.9)	1.1 ± 0.3 (1.2)	0.07	1.0 ± 0.2 (1.1)	1.2 ± 0.3 (1.3)	0.09
SPPB (scores)	9.1 ± 2.3 (10.0)	10.1 ± 1.2 (10.0) *	0.41	10.8 ± 1.1 (11.0)	11.2 ± 1.3 (12.0)	0.21
Sarcopenia (*n*, %) ^6^	6 (33.3%)	3 (30.0%)	1.00	1 (11.1%)	3 (10.0%)	1.00
	Female					
	Elderly ^4^			Middle-Age ^4^		
	OA (*N* = 56)	Non-OA (*N* = 23)	*p* Value ^5^	OA (*N* = 17)	Non-OA (*N* = 37)	*p* Value ^5^
Muscle mass						
Whole body muscle mass (kg)	37.4 ± 5.6 (38.1) *	35.3 ± 6.3 (34.2) *	0.03	42.4 ± 7.4 (40.6)	40.2 ± 7.4 (39.3)	0.32
Trunk muscle mass (kg)	19.2 ± 3.2 (19.2) *	17.7 ± 2.6 (17.4) *	0.05	22.0 ± 3.7 (22.3)	20.4 ± 3.7 (19.7)	0.13
WSMI (kg/m^2^)	16.1 ± 2.2 (16.2)	14.9 ± 2.0 (14.9) *	0.03	17.1 ± 2.5 (17.2)	16.3 ± 2.6 (16.2)	0.29
ASMI (kg/m^2^)	6.5 ± 1.0 (6.6)	6.2 ± 1.3 (6.0)*	0.06	6.9 ± 1.2 (7.2)	6.8 ± 1.2 (6.7)	0.69
Muscle strength and endurance						
Handgrip strength (kg)	19.3 ± 4.1 (19.4) *	19.8 ± 5.5 (19.8)	0.83	23.0 ± 4.5 (23.9)	22.0 ± 3.8 (21.9)	0.43
Dumbbells curls (reps)	14.5 ± 3.7 (15.0) *	14.5 ± 5.4 (13.5) *	0.95	18.5 ± 4.8 (18.0)	17.1 ± 3.4 (17.0)	0.25
Leg-back strength (kg)	36.2 ± 12.8 (34.8) *	36.1 ± 14.0 (35.0) *	0.92	45.4 ± 15.7 (48.5)	46.7 ± 13.9 (48.0)	0.76
Chair-stand test (reps)	10.4 ± 3.8 (10.0) *	10.6 ± 4.0 (10.0) *	0.79	13.2 ± 4.1 (14.0)	13.5 ± 3.7 (13.0)	0.93
Gait speed (m/s)	0.8 ± 0.3 (0.9) *	0.9 ± 0.2 (1.0) *	0.40	1.0 ± 0.2 (1.0)	1.1 ± 0.2 (1.1)	0.10
SPPB (scores)	9.1 ± 2.2 (9.0) *	9.6 ± 1.9 (9.0) *	0.25	10.8 ± 1.7 (11.0)	11.0 ± 1.0 (11.0)	0.85
Sarcopenia (*n*, %) ^6^	8 (14.3%)	6 (26.1%)	0.33	2 (11.8%)	4 (10.8%)	1.00

^1^ Means ± SD (medians). ^2^ Comparison between OA and Non-OA groups. ^3^ Comparison between gender in the OA or Non-OA group. † Comparison between OA or Non-OA in the same gender group, *p* < 0.05. ^4^ Elderly: ≥65 y; middle-age: 40–64 y. ^5^ Comparison between OA or Non-OA in the same age group. * Comparison between different age groups with OA or without OA, *p* < 0.05. ^6^ Sarcopenia was defined according to the Asian Working Group. ASMI, appendicular skeletal muscle mass index; BMI, body mass index; BUN, blood urea nitrogen; GOT, glutamic-oxaloacetic transaminase; GPT, glutamate-pyruvate transaminase; hs-CRP, high sensitivity-C reactive protein; OA, osteoarthritis; SPPB, short physical performance battery; WSMI, whole skeletal muscle mass index.

**Table 2:** Coenzyme Q10 status, oxidative stress, and antioxidant capacity of the subjects.

	Elderly ^2^			Middle-Age ^2^		
	OA (*N* = 74)	Non-OA (*N* = 33)	*p* Value ^3^	OA (*N* = 26)	Non-OA (*N* = 67)	*p* Value ^3^
Coenzyme Q10 (µM) ^1^	0.41 ± 0.15 (0.41)	0.47 ± 0.22 (0.46)	0.18	0.44 ± 0.13 (0.42)	0.47 ± 0.18 (0.43)	0.44
Coenzyme Q10/TC (µmol/mmol)	0.08 ± 0.03 (0.08)	0.10 ± 0.04 (0.09)	0.06	0.09 ± 0.03 (0.09)	0.09 ± 0.03 (0.09)	0.99
Oxidative stress						
MDA (µM)	2.6 ± 0.5 (2.5)	2.7 ± 0.6 (2.6)	0.29	2.5 ± 0.4 (2.5)	2.7 ± 0.5 (2.7)	0.09
Protein carbonyl (nmole/mg protein)	0.38 ± 0.06 (0.37)	0.38 ± 0.07 (0.38) *	0.96	0.36 ± 0.05 (0.37)	0.34 ± 0.07 (0.33)	0.02
Antioxidant capacity						
Serum TAC (mM Trolox)	5.8 ± 0.5 (5.7)	5.4 ± 0.3 (5.4)	<0.01	5.9 ± 0.4 (6.0)	5.4 ± 0.3 (5.5)	<0.01
RBC TAC (mM Trolox)	10.2 ± 0.7 (10.1) *	10.0 ± 0.5 (10.0)	0.16	10.6 ± 0.7 (10.4)	10.1 ± 0.5 (10.0)	<0.01

^1^ Means ± SD (medians). ^2^ Elderly: ≥65 y; middle-age: 40–64 y. ^3^ Comparison between OA and non-OA in the same age group. * Comparison between different age groups with OA or without OA, *p* < 0.05. MDA, malondialdehyde; OA, osteoarthritis; RBC, red blood cell; TAC, total antioxidant capacity; TC, total cholesterol.

**Table 3:** Correlation between coenzyme Q10 status and oxidative stress, muscle mass, muscle strength, and muscle endurance stratified by age.

	Coenzyme Q10/TC (µmol/mmol)			
	Elderly ^2^		Middle-Age ^2^	
	OA (*N* = 74)	Non-OA (*N* = 33)	OA (*N* = 26)	Non-OA (*N* = 67)
Oxidative stress				
MDA (µM)	−0.21 ^1,#^	0.18	−0.07	0.09
Protein carbonyl (nmole/mg protein)	−0.12	−0.27	−0.08	−0.11
Antioxidant capacity				
Serum TAC (mM Trolox)	0.20 ^†^	−0.10	0.19	−0.02
RBC TAC (mM Trolox)	0.26 *	−0.19	0.20	0.13
Muscle mass				
Whole body muscle mass (kg)	0.21 ^†^	0.15	0.20	0.30 *
Trunk muscle mass (kg)	0.15	0.25	0.30	0.28 *
WSMI (kg/m^2^)	0.22 ^#^	0.15	0.27	0.36 **
ASMI (kg/m^2^)	0.26 *	0.08	0.10	0.38 **
Muscle strength and endurance				
Handgrip strength (kg)	0.19	0.04	0.17	0.18
Dumbbells curls (reps)	0.24 *	0.24	−0.01	0.20
Leg-back strength (kg)	0.29 *	0.19	0.25	0.27 *
Chair-stand test (reps)	0.03	0.33 ^‡^	0.17	0.12
Gait speed (m/s)	0.33 **	0.17	0.31	0.05
SPPB (scores)	0.16	0.28	0.12	0.15

^1^ Correlation coefficients. * *p* < 0.05, ** *p* < 0.01, ^‡^ *p* = 0.06, ^#^ *p* = 0.07, ^†^ *p* = 0.08. ^2^ Elderly: ≥65 y; middle-age: 40–64 y. ASMI, appendicular skeletal muscle mass index; MDA, malondialdehyde; OA, osteoarthritis; SPPB, short physical performance battery; TAC, total antioxidant capacity; TC, total cholesterol; WSMI, whole skeletal muscle mass index.

**Table 4:** Correlations between oxidative stress, antioxidant capacity, muscle mass, muscle strength, and muscle endurance of the subjects.

	Oxidative Stress		Antioxidant Capacity	
	MDA (µM)	Protein Carbonyl (nmole/mg protein)	Serum TAC (mM Trolox)	RBC TAC (mM Trolox)
OA + Non-OA (*N* = 200)				
Whole body muscle mass (kg)	0.11 ^1^	−0.23 **	0.01	0.09
Trunk muscle mass (kg)	0.08	−0.26 **	0.04	0.10
WSMI (kg/m^2^)	0.08	−0.25 **	0.07	0.15 *
ASMI (kg/m^2^)	0.13	−0.22 **	0.04	0.10
Handgrip strength (kg)	0.13	−0.07	−0.13	−0.03
Dumbbells curls (reps)	−0.04	−0.13	−0.06	−0.02
Leg-back strength (kg)	0.10	−0.15 *	−0.03	0.04
Chair-stand test (reps)	−0.01	−0.13	−0.03	−0.00
Gait speed (m/s)	0.14	−0.33 **	0.12	0.27 **
SPPB (scores)	0.01	−0.26 **	0.01	0.11
Elderly ^2^ OA + Non-OA (*N* = 107)				
Whole body muscle mass (kg)	0.12	−0.12	0.02	0.08
Trunk muscle mass (kg)	0.13	−0.14	0.04	0.09
WSMI (kg/m^2^)	0.08	−0.17	0.09	0.14
ASMI (kg/m^2^)	0.09	−0.14	0.10	0.10
Handgrip strength (kg)	0.06	0.04	−0.17	−0.03
Dumbbells curls (reps)	−0.03	−0.02	−0.06	−0.00
Leg-back strength (kg)	−0.00	0.01	−0.04	0.09
Chair-stand test (reps)	−0.03	−0.04	−0.05	−0.02
Gait speed (m/s)	0.09	−0.10	0.17	0.38 **
SPPB (scores)	0.00	−0.10	−0.02	0.08
Middle-age ^2^ OA + Non-OA (*N* = 93)				
Whole body muscle mass (kg)	0.08	−0.23 *	0.03	0.01
Trunk muscle mass (kg)	0.02	−0.26 *	0.08	0.05
WSMI (kg/m^2^)	0.07	−0.23 *	0.07	0.11
ASMI (kg/m^2^)	0.17	−0.20	−0.01	0.04
Handgrip strength (kg)	0.17	0.01	−0.08	−0.11
Dumbbells curls (reps)	−0.12	−0.08	−0.08	−0.12
Leg-back strength (kg)	0.21*	−0.17	−0.03	−0.09
Chair-stand test (reps)	−0.04	−0.02	−0.01	−0.10
Gait speed (m/s)	0.15	−0.42 **	0.12	0.12
SPPB (scores)	−0.04	−0.23 *	0.10	0.07

^1^ Correlation coefficients. * *p* < 0.05 and ** *p* < 0.01. ^2^ Elderly: ≥65 y; middle-age: 40–64 y. ASMI, appendicular skeletal muscle mass index; MDA, malondialdehyde; OA, osteoarthritis; SPPB, short physical performance battery; TAC, total antioxidant capacity; WSMI, whole skeletal muscle mass index.

## Abbreviations

ASMI	appendicular skeletal muscle mass index
BMI	body mass index
BUN	blood urea nitrogen
GOT	glutamic oxaloacetic transaminase
GPT	glutamic pyruvic transaminase
HPLC	high-performance liquid chromatography
Hs-CRP	high-sensitivity C-reactive protein
K/L	Kellgren and Lawrence
MDA	Malondialdehyde
OA	Osteoarthritis
RBC	red blood cell
ROS	reactive oxygen species
SPPB	short physical performance battery
TAC	Total antioxidant capacity
TC	total cholesterol
WSMI	whole skeletal muscle mass index
